# Elevated Anti-Müllerian Hormone as a Prognostic Factor for Poor Outcomes of In Vitro Fertilization in Women with Polycystic Ovary Syndrome

**DOI:** 10.3390/biomedicines11123150

**Published:** 2023-11-27

**Authors:** Emídio Vale-Fernandes, Márcia Barreiro, Carla Leal, Rosa Zulmira Macedo, António Tomé, Mariana P. Monteiro

**Affiliations:** 1 Centre for Medically Assisted Procreation/Public Gamete Bank, Gynaecology Department, Centro Materno-Infantil do Norte Dr. Albino Aroso (CMIN), Centro Hospitalar Universitário de Santo António (CHUdSA), 4050-651 Porto, Portugal; diretora.pma@chporto.min-saude.pt (M.B.); carlaleal.pma@chporto.min-saude.pt (C.L.); 2 UMIB—Unit for Multidisciplinary Research in Biomedicine, ICBAS—School of Medicine and Biomedical Sciences, University of Porto, 4050-313 Porto, Portugal; mpmonteiro@icbas.up.pt; 3 ITR—Laboratory for Integrative and Translational Research in Population Health, 4050-313 Porto, Portugal; 4 Gynaecology Department, Centro Materno-Infantil do Norte Dr. Albino Aroso (CMIN), Centro Hospitalar Universitário de Santo António (CHUdSA), 4050-651 Porto, Portugal; diretora.ginecologia@chporto.min-saude.pt (R.Z.M.); antoniotome.dm@chporto.min-saude.pt (A.T.)

**Keywords:** in vitro fertilization (IVF), anti-Müllerian hormone (AMH), polycystic ovary syndrome (PCOS), live birth rate, cumulative live birth rate

## Abstract

Women with polycystic ovary syndrome (PCOS) tend to have elevated anti-Müllerian hormone (AMH) levels, which appear to correlate with disease severity and pregnancy outcomes. This was a retrospective observational study designed to assess the relationship between circulating AMH levels and in vitro fertilization (IVF) outcomes. The study involved 150 women with PCOS who underwent IVF treatments. The women’s IVF cycles were allocated into three subgroups according to AMH levels: ‘low’ (AMH < 3.7 ng/mL; *n* = 49), ‘middle’ (AMH 3.7–7.4 ng/mL; *n* = 94), and ‘high’ (AMH > 7.4 ng/mL; *n* = 56). All pregnancy-related outcomes (positive beta human chorionic gonadotropin (βHCG), clinical pregnancy rate, live birth rate, and cumulative live birth rate) were greater in women’s IVF cycles with ‘low’ AMH when compared to those with ‘middle’ or ‘high’ AMH (*p* < 0.05). AMH levels below 3.7 ng/mL were found to be associated with lower oocyte immaturity rate and better pregnancy outcomes, although baseline AMH was not shown to have any significant predictive power for live birth and cumulative live birth in the multivariable logistic regression analysis after adjusting for possible confounders nor in the ROC analyses. In summary, the current study lays the groundwork to validate high AMH levels as a poor prognostic factor for pregnancy outcomes after IVF in women with PCOS.

## 1. Introduction

Polycystic ovary syndrome (PCOS) is a common endocrine disorder in women of reproductive age with a prevalence of 5 to 18% [1]. PCOS is characterized by amenorrhea or oligomenorrhea, excessive androgen production leading to clinical signs of hyperandrogenism, such as hirsutism, and the presence of polycystic ovaries [2]. Moreover, uncontrolled steroidogenesis, abnormal insulin sensitivity, and obesity are features frequently associated with this disorder [3].

Anovulatory infertility is often observed as a manifestation of PCOS [4]. Whenever ovulation induction treatments prove to be unsuccessful in women with PCOS, in vitro fertilization (IVF) or intracytoplasmic sperm injection (ICSI) may be considered as infertility therapy [5]. In the past few years, several markers have been appointed as able to predict successful pregnancy after assisted reproductive technology (ART) [6,7]. Women’s age, body mass index (BMI), antral follicle count (AFC), serum concentrations of anti-Müllerian hormone (AMH), and follicle-stimulating hormone (FSH) measured on the 3rd day of the menstrual cycle, are among the most robust markers identified [8].

AMH is a glycoprotein synthesized by granulosa cells of preantral and small antral follicles, and involved in granulosa cell growth and differentiation [9]. AMH inhibits the recruitment of primordial follicles and regulates the growth and development of follicles [10]. In healthy women, AMH circulating levels remain stable along the menstrual cycle [11]. Since AMH acts as a surrogate of ovarian reserve, it can be used as a prognosis biomarker in women undergoing IVF [12,13]. Meczekalszi et al. suggested that AMH could be used as a decision aid in the selection of the ovarian stimulation protocol, in order to improve the treatment outcomes in women of late reproductive age [14]. According to these authors, AMH can be considered as a biomarker of fertility and predictor of pregnancy outcomes in assisted reproductive technology cycles. However, AMH use as prognostic marker in IVF is not universally accepted, since opposite findings were also reported [15].

The predictive value of AMH is particularly controversial in women with PCOS, whose levels are volatile [15]. Women with PCOS tend to have elevated AMH levels, which appear to correlate with disease severity and may affect AMH’s predictive value to assess ovarian reserve, thus also impacting on AMH’s predictive value for pregnancy outcomes. Of note, there are few studies reporting on the relationship between AMH and reproductive outcomes in women with PCOS, which yield contradictory findings, with some suggesting a positive relationship [16,17], while others suggesting the opposite [15,18], highlighting the need for further research in order to better understand the complex relationship between AMH levels and PCOS.

The aim of this retrospective study was to investigate the relationship between AMH levels and IVF outcomes in women with PCOS, with live birth rate and cumulative live birth rate as the primary outcomes. This study was designed in order to provide further insights into the prognostic value of AMH levels in women with PCOS undergoing IVF and as a decision aid in selecting the most appropriate treatment strategy.

## 2. Materials and Methods

### 2.1. Participants

The study population comprised women with PCOS (*n* = 150) who underwent IVF/ICSI cycles with autologous oocytes (*n* = 199), at a single academic public center between 1 January 2018 and 31 December 2022. For inclusion, women had to have a PCOS diagnosis according to the Rotterdam criteria established in 2003, through the presence of at least two of the three following characteristics: clinical and/or biochemical signs of hyperandrogenism, anovulation (oligo- or amenorrhea), and/or polycystic ovaries on ultrasound. A presumptive diagnosis of PCOS without fulfilling the Rotterdam criteria or clinical or biochemical hyperandrogenism due to causes other that PCOS were considered exclusion criteria. Electronic medical records were used for data extraction, namely, age, body mass index (BMI) calculated using the formula weight (kilogram)/height^2^ (meter), hormonal measurements, and IVF cycle characteristics. The study protocol was authorized by the Ethics Committee of the Institution (Reference—2020.119(097-DEFI/099-CE)).

The women’s IVF cycles were categorized into three groups based on baseline AMH serum levels: ‘low’ AMH comprising IVF cycles of women with AMH levels below the 25th percentile (AMH < 3.7 ng/mL; *n* = 49), ‘middle’ AMH comprising IVF cycles of women with AMH levels between the 25th and 75th percentile (3.7–7.4 ng/mL; *n* = 97), and ‘high’ AMH comprising IVF cycles of women with AMH levels above the 75th percentile (AMH > 7.4 ng/mL, *n* = 54). The classification terms ‘low’, ‘middle’, and ‘high’ are used for simplified description and comparative purposes only, and they are not intended to be synonymous with biological normality. Baseline AMH does not follow a normal distribution.

### 2.2. Ovarian Stimulation and IVF Procedures

Women underwent an GnRH antagonist protocol with individualized controlled ovarian stimulation based on ovarian reserve testing and standard clinical practice. On day 3, one or two embryos with the highest score were selected for transfer, while any remaining embryos were either cryopreserved or allowed to continue development to the blastocyst stage for fresh-embryo transfer or cryopreservation. The decision to freeze all embryos was made based on local criteria, which included factors such as the patient’s risk of ovarian hyperstimulation syndrome (OHSS), unsuitable endometrial environment, premature progesterone elevation, or personal circumstances that favored frozen-embryo transfer over fresh-embryo transfer.

### 2.3. Primary Assessment Measures

The primary outcomes included live birth rate (LBR) and cumulative live birth rate (CLBR). Live birth was defined as delivery of one or more live infants. LBR was defined as the number of live births divided by the number of women in a group. CLBR was defined as live birth that occurs during the fresh IVF cycle and the subsequent frozen-embryo transfer cycle(s) after a single ovarian stimulation IVF cycle until one live birth. Clinical pregnancy was defined as the visualization of one or more gestational sacs on ultrasonography.

### 2.4. Statistical Analysis

Data entry was performed using IBM SPSS Statistics 25, Armonk, NY, USA. A statistical significance level of 0.05 was used. The primary analysis of the study involved comparing the main baseline characteristics, IVF/ICSI cycle characteristics, and pregnancy outcomes among the three groups. Categorical data were presented as percentages and the number of cases, while continuous data were reported as mean ± standard deviation (SD).

For categorical variables, comparisons were made using the χ^2^ test. Continuous variables were assessed for normal distribution and homogeneity of variance. In cases where the data did not meet these assumptions (non-normal distribution or heterogeneity of variance), the Kruskal–Wallis test was employed.

The degree of association of AMH levels and reproductive outcomes of women with PCOS was calculated and evaluated by Spearman’s rank correlation. Binomial logistic regression analysis models were performed to adjust for the effect of various confounders on numerical and categorical outcomes, respectively. The receiver operating characteristic (ROC) curves for variables were created to explore the predictive power of baseline AMH for live birth and cumulative live birth. Missing values represented less than 5% per variable; they were missing completely at random, and therefore these values were not replaced.

## 3. Results

Women with PCOS had a mean age of 32.95 years (±3.87) and a mean BMI of 25.47 kg/m^2^ (±5.87). Among the assisted reproduction treatments, 43.1% of the women underwent ICSI, 31.6% underwent IVF, and 14.7% underwent both treatments. On day 3 of the menstrual cycle, the mean levels of LH and FSH were 8.22 ± 3.90 and 6.23 ± 1.54 mUI/mL, respectively. The mean (±SD) value of baseline serum AMH was 6.28 ± 3.72 ng/mL.

The IVF cycles (*n* = 199) were divided according to the AMH levels. Women with PCOS and lower AMH serum levels tended to be older and have a lower BMI, although not statistically different (*p* > 0.05). Moreover, no statistical differences (*p* > 0.05) were found between groups for the other baseline characteristics and for the infertility factors. Women with higher AMH levels had higher LH levels and lower FSH levels; the LH:FSH ratio was greater than 1 in the three groups (‘low’ versus ‘high’: *p* = 0.013, ‘middle’ versus ‘high’: *p* = 0.006). Table 1 presents the baseline IVF cycle characteristics according to the AMH levels.

The IVF cycle characteristics and outcomes are presented in Table 2. Women with ‘low’, ‘middle’, and ‘high’ AMH levels underwent a mean of 1.25 ± 0.56, 1.31 ± 0.59, and 1.21 ± 0.46 follicular punctures for oocyte retrieval, respectively. No significant differences were found between groups for these characteristics. Among the analyzed IVF cycles, the mode of fertilization (IVF or ICSI) significantly differed between the AMH groups. The IVF cycles with ‘middle’ AMH levels presented a higher percentage of ICSI cycles when compared with the other AMH groups (*p* = 0.023). The total dose of FSH administered decreased progressively from ‘low’ to ‘high’ AMH levels (‘low’ versus ‘middle’: *p* = 0.004, ‘low’ versus ‘high’: *p* = 0.027). Nevertheless, the number of days of stimulation did not differ. The number of cumulus–oocyte complexes (COCs) and follicle count at trigger were similar in the three groups. The oocyte immaturity rate was lower in the ‘low’ AMH group (*p* = 0.002). The number of two pronucleated oocytes was higher in the ‘high’ AMH group and lower in the ‘middle’ AMH group. No differences between the AMH groups were found regarding the fertilization rate, cleavage rate, number of obtained embryos, and number of cryopreserved embryos. Although the number of IVF cycles with embryo freezing did not differ among women with different AMH levels, the percentage of IVF cycles where the ‘freeze all’ strategy was applied almost doubled in women with ‘high’ AMH when compared to that of women with ‘low’ AMH. The proportion of fresh-embryo transfer was higher in the ‘low’ AMH group and lower in the ‘high’ AMH group (*p* = 0.004). No differences between the AMH groups were found regarding the proportion of frozen-embryo transfer.

All pregnancy-related outcomes (positive beta human chorionic gonadotropin—βHCG, clinical pregnancy rate, live birth rate, and cumulative live birth rate) were significantly higher in women with ‘low’ AMH in comparison to women with ‘middle’ or ‘high’ AMH levels.

A positive correlation between serum AMH concentrations and BMI, LH serum levels and LH:FSH ratio at day 3, and follicle count at trigger was found (Table 3). An inverse correlation was found between serum AMH levels and age, FSH serum levels, and total dose of FSH administered. Serum AMH concentrations did not differ with the number of COCs. There was no statistically significant correlation between circulating AMH and clinical pregnancy rate (r, −0.129, *p* = 0.076), live birth rate (r, −0.114; *p* = 0.119), or cumulative live birth rate (r, −0.133; *p* = 0.068).

**Figure 1:**
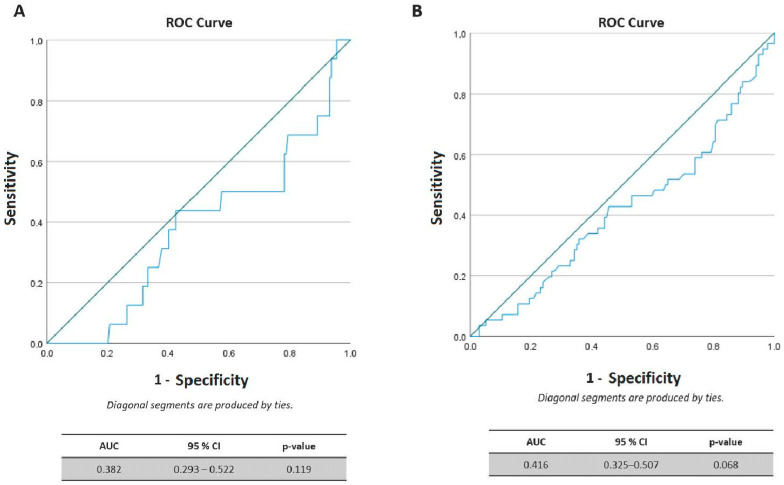
The receiver operating characteristic (ROC) curve analysis for AMH as a predictor of (**A**) live birth rate (LBR) and (**B**) cumulative live birth rate (CLBR), denoted overall poor predictive value of AMH for pregnancy outcomes.

A binomial logistic regression analysis was conducted to adjust for confounders’ effect on live birth. The model included age, BMI, FSH, and AMH as independent variables. No significant association was revealed between AMH and live birth (*p* = 0.104) or cumulative live birth (*p* = 0.175). Similarly, the predictive power of baseline AMH for live birth and cumulative live birth was explored using ROC curves (Figure 1). The area under the curve (AUC) of 0.382 (0.293–0.522, 95% confidence interval, *p* = 0.119) for live birth and 0.416 (0.325–0.507, 95% confidence interval, *p* = 0.068) for cumulative live birth indicates overall poor predictive value.

## 4. Discussion

The present retrospective study in women with PCOS explored the impact of serum AMH levels on IVF-related outcomes, namely, live birth rate and cumulative live birth rate. An inverse correlation between AMH levels and clinical pregnancy rate, live birth rate, and cumulative live birth rate was found in PCOS women. Women with PCOS and lower AMH levels achieved higher rates of live birth and cumulative live birth and had lower number of IVF cycles with a ‘freeze all’ strategy.

Importantly, our study showed that women with PCOS undergoing assisted reproduction with ‘high’ AMH had significantly lower rates of clinical pregnancy, live birth, and cumulative live birth when compared to those with ‘middle’ or ‘low’ AMH levels. Nevertheless, by performing a multivariable logistic regression analysis in a forward manner, and after adjusting for possible confounders, no significant relationship was found. These results were further confirmed by the ROC curve analysis which also denoted the poor predictive value of baseline AMH levels.

Of note, several studies have explored the relationship between baseline serum AMH levels and pregnancy outcomes following IVF/ICSI treatment [19,20,21]. However, in women with PCOS, results have been inconsistent. While some authors reported higher AMH levels to be associated with better outcomes [18,22], others found the opposite [23,24]. Our results are in line with Xi et al. [18], who found the lowest clinical pregnancy rate in women with PCOS and high AMH levels, and with Tal et al. [25], who reported that higher AMH levels were associated with lower live birth rates in fresh IVF cycles. These disparities could be attributed to intrinsic differences between study populations and sample sizes. Our results add another piece of evidence, by suggesting that lower AMH levels significantly contribute to better IVF outcomes in women with PCOS.

In addition, AMH levels appear to be a relevant marker of PCOS severity, with elevated levels suggesting severe disease and masking or confounding the predictive effect on ovarian reserve and pregnancy outcomes [15,17,22,26]. The oocyte immaturity rate was lower in the ‘low’ AMH group, although no differences between the AMH groups were found regarding the fertilization rate, cleavage rate, number of obtained embryos, and number of cryopreserved embryos. The current study lays the groundwork to validate AMH levels as a prognostic factor for pregnancy after IVF in women with PCOS, as AMH levels below 3.7 ng/mL were found to be associated with better pregnancy outcomes. This range of AMH values (under 3.5 ng/mL) is found among healthy women and has been linked with good ovarian response to stimulation [27,28,29]. Although this seems to be transversal to PCOS, the relationship between baseline AMH levels and pregnancy outcomes in women with PCOS may not be as straightforward and requires further investigation. Elevated LH, testosterone, and AMH levels are hallmarks of gonadotrophic axis dysregulation associated with PCOS; indeed, several independent studies documented higher AMH levels in the follicular fluid composition of women with PCOS when compared with unaffected controls [30].

Additionally, although age distribution was not significantly different among our study groups, AMH levels are known to decrease with age [31,32]. Moreover, according Rivelli et al. (2016), in patients with very low serum AMH, IVF results are significantly affected by chronological age, since women under 35 years old experience a higher number of oocytes retrieved and clinical pregnancy rate than older women, despite comparable AMH levels [33]. This is a well-known factor, since as a woman ages there is a decline in the number of primordial follicles, accompanied by a concomitant decrease in AMH serum levels [34]. Nevertheless, in women with PCOS, due to the pathophysiology of the disorder, AMH positively correlates with the severity of PCOS phenotype rather than age [22,26,35].

In contrast, although BMI was numerically higher in women with ‘high’ AMH levels, the difference was not statistically significant. In fact, there is a controversial relationship between AMH levels and BMI, with some studies suggesting a negative correlation [36,37], while others showing either no relationship or a positive correlation [38,39,40].

Our study found an inverse relationship between FSH and AMH levels and a positive trend between LH and AMH in women with PCOS, in accordance with previous studies [18,23,26,41,42]. However, despite the fact that the LH/FSH ratio has been used for establishing the diagnosis of PCOS [43,44], little is known about its predictive value for IVF outcomes. Nevertheless, the total dose of gonadotrophins administered was significantly lower in women with PCOS and ‘high’ compared to ‘low’ AMH levels. Our findings are consistent with previous studies that suggest that AMH could be useful to predict ovarian responsiveness to gonadotrophins [45,46]. These results are somehow counterintuitive, considering that AMH was shown to inhibit FSH-stimulated aromatase mRNA expression in human granulosa-luteal cells, implying that higher AMH levels may result in reduced follicle sensitivity to FSH and decreased estradiol production [45].

Moreover, although there was no difference in the number of retrieved follicles among the three groups, women with higher AMH levels had a higher number of two pronucleated oocytes and IVF cycles with a ‘freeze all’ strategy. The ‘freeze-all’ strategy, i.e., the cryopreservation of all mature oocytes or viable embryos after ovarian stimulation for transferring embryos in a later natural or artificial cycle to an endometrium that has not been exposed to high doses of exogenous gonadotropins, has been hypothesized to improve IVF outcomes by increasing clinical pregnancy rate while avoiding ovarian hyperstimulation syndrome (OHSS) [47,48,49]. At first sight, this conjugation of factors could be propelling the cumulative live birth in women with higher AMH levels. However, our results are in line with a recent meta-analysis which concluded that “there is probably little or no difference in cumulative live birth rate between the ‘freeze all’ strategy and the conventional IVF/ICSI strategy” [49]. The main widely recognized advantage for the ‘freeze all’ strategy is the prevention of OHSS, which is more frequent in women with PCOS and ‘high’ AMH levels [50]. Nevertheless, our data cannot support that hypothesis nor strategy.

Despite providing a relevant contribution to knowledge on the relationship between AMH levels and IVF outcomes in women with PCOS, with the major strength of being focused on live birth rates, the gold standard outcome for fertility studies, our study harbors some limitations that must be acknowledged. The major limitation is its retrospective design. Even though the primary and secondary outcomes are objective measures, which minimize the impact of subjective factors in patient assessment, there are inevitable residual confounding factors that cannot be avoided, such as subtle modifications of the ovarian stimulation protocols and other unidentified confounding factors, which may have affected the study outcomes. Additionally, the retrospective analysis was limited to data previously available, while in a prospective study, other specified parameters, such as androgen levels and genotyping, could have been collected. Androgen levels could be useful not only for detailed phenotypic characterization, but also to explore the correlation between androgens and AMH, while gene polymorphisms could contribute to explain the phenotypic variability and provide further insights on the expected outcomes [51,52]. Another limitation is the fact that the endometrium was not evaluated. Despite the fact that PCOS-related infertility has been traditionally attributed to chronic anovulation, a defective endometrium secondary to high androgens and AMH, oxidative stress, and inflammation could be responsible for implantation failure and recurrent miscarriage, and therefore be accountable for infertility in women with PCOS [53,54,55].

Due to the fact that our study was conducted at a public center, where IVF treatments are restricted to women under 40 years old and avoided in women with morbid obesity, the women under study tend to be clustered within a relatively narrow age and BMI interval, precluding the conduction of stratified data analysis according to chronological age or BMI. Additionally, since a preimplantation genetic test is not conducted at our center, and no embryo transfer is conducted at weekends or bank holidays, embryo transfer does not always take place at the blastocyst stage, which is considered the gold standard practice in the sense of optimizing quality embryo selection. Finally, the relatively small sample, comprising 150 women with PCOS who underwent 199 IVF cycles, could also have been responsible for statistically significant differences that went unnoticed.

In sum, the present study confirms that high AMH levels may impair the ovarian response in women with PCOS undergoing IVF, and could be of clinical utility to predict treatment outcomes, as recently shown by a systematic review and meta-analysis which suggested that AMH could be useful for counseling women with PCOS, who wish to undergo fertility treatments, on the expected IVF outcomes, although large-scale, high-quality cohort studies are still needed to confirm these findings [56]. To gain a deeper understanding on the impact of AMH on folliculogenesis and the oocyte quality of women with PCOS, additional clinical studies and molecular biology experiments are needed. Furthermore, future studies exploring fertilization, blastulation, and implantation rates could provide relevant information on oocyte quality in women with PCOS.

## 5. Conclusions

Overall, our findings uphold the hypothesis that in women with PCOS, ‘high’ AMH levels may have a negative impact on clinical pregnancy rate, live birth rate, and cumulative live birth rate. These findings bring into consideration that in women with PCOS, high AMH levels, instead of reflecting the ovarian reserve, act as a biomarker of disease severity and worse reproductive prognosis. 

## Figures and Tables

**Table 1:** Baseline reproductive characteristics and hormone levels of in vitro fertilization (IVF) cycles according to the anti-Müllerian hormone (AMH) levels.

	Low AMH (<3.7 ng/mL) (*n* = 49)	Middle AMH (3.7–7.4 ng/mL) (*n* = 94)	High AMH (AMH > 7.4 ng/mL) (*n* = 54)	*p*-Value
Age (years) [minimum (22), maximum (39)]	33.83 ± 3.91	32.97 ± 3.57	32.47 ± 3.92	NS ^a^
Weight (kg)	66.15 ± 11.70	66.23 ±14.65	69.46 ± 13.02	NS ^a^
Height (cm)	162.2 ± 7.13	162.4 ± 6.89	163.3 ± 6.98	NS ^a^
BMI (kg/m^2^)	24.61 ± 4.31	24.44 ± 4.22	26.51 ± 4.95	NS ^a^
Primary infertility type [*n* (%)]	43 (87.8)	70 (74.5)	47 (87.0)	NS ^d^
Smoker [*n* (%)]	5 (10.2)	11 (11.7)	6 (11.1)	NS ^d^
PCOS + other infertility factor [*n* (%)]	24 (49.0)	42 (44.7)	30 (55.6)	NS ^d^
Male infertility factor [*n* (%)]	35 (71.4)	77 (81.9)	41 (75.9)	NS ^d^
Duration of infertility (months)	40.06 ± 19.92	45.02 ± 22.97	46.37 ± 24.31	NS ^a^
LH, mUI/mL	7.39 ± 2.90	7.88 ± 2.58	9.37 ± 4.38	NS ^b^
FSH, mUI/mL	6.65 ± 1.44	6.58 ± 1.23	5.58 ± 1.20	<0.001 ^b,c^
LH:FSH ratio	1.25 ± 0.60	1.26 ± 0.59	1.64 ± 0.83	0.013 ^b^, 0.006 ^c^
E2, pg/mL	43.43 ± 17.90	40.77 ± 14.51	39.30 ± 15.67	NS ^a^

Data expressed as mean ± standard deviation. *p*-value < 0.05 was considered statistically significant. BMI: body mass index; E2: estradiol; FSH: follicle-stimulating hormone; LH: luteinizing hormone; PCOS: polycystic ovary syndrome. ^a^ The Kruskal–Wallis multiple comparison test was used to determine which group differed from others. ^b^ Comparison between low and high. ^c^ Comparison between middle and high. ^d^ Chi-square test. NS: not statistically different.

**Table 2:** In vitro fertilization (IVF) cycle characteristics and assisted reproductive technology (ART) outcomes.

	Low AMH (AMH < 3.7 ng/mL) (*n* = 49)	Middle AMH (3.7–7.4 ng/mL) (*n* = 94)	High AMH (AMH > 7.4 ng/mL) (*n* = 54)	*p*-Value
Intracytoplasmic sperm injection (ICSI), %	17 (34.7)	57 (60.1)	23 (42.6)	0.023 ^d^
Total FSH dose, UI	2 545 ± 488	2 083 ± 644	2 030 ± 680	0.004 ^a,b^, 0.027 ^a,c^
Number of stimulation days	11.00 ± 1.77	9.92 ± 1.72	10.58 ± 2.12	NS ^a^
No. of follicles	7.16 ± 5.88	7.70 ± 4.89	8.18 ± 6.22	NS ^a^
No. of COCs	15.54 ± 9.38	16.08 ± 6.58	19.29 ± 7.08	NS ^a^
Oocyte immaturity rate (%)	13.19 ± 12.14	22.38 ± 17.03	18.63 ± 18.16	0.002 ^a,c^
No. of two pronucleated oocytes	7.04 ± 3.80	5.82 ± 3.76	7.94 ± 3.58	0.045 ^a,b^, 0.032 ^a,c^
Fertilization rate (%)	59.56	57.18	63.34	NS ^a^
Cleavage rate (%)	92.53	95.80	98.00	NS ^a^
No. of obtained embryos	3.50 ± 2.62	2.84 ± 2.80	3.63 ± 2.70	NS ^a^
Fresh-embryo transfer (%)	38.98	33.24	15.38	0.004 ^a,b^
No. of IVF cycles with embryo freezing [*n* (%)]	29 (59.2)	53 (56.4)	37 (66.1)	NS ^d^
No. of IVF cycles with freeze all [*n* (%)]	15 (30.6)	48 (51.1)	34 (60.7)	0.007 ^d^
No. of cryopreserved embryos	2.66 ± 2.72	2.44 ± 3.10	3.33 ± 2.89	NS ^a^
Frozen-embryo transfer (%)	22.32	34.28	32.58	NS ^a^
No. of FET per IVF cycle				0.005 ^d^
1	18 (54.5)	44 (83.0)	26 (68.4)	
2	9 (27.3)	6 (11.3)	5 (13.2)	
3	1 (3.0)	2 (3.8)	6 (15.8)	
Positive βHCG [*n* (%)]	17 (34.7)	13 (13.8)	6 (11.1)	0.002 ^d^
Clinical pregnancy rate [*n* (%)]	12 (24.5)	11 (11.7)	3 (5.6)	0.013 ^d^
Live birth rate [*n* (%)]	10 (20.4)	6 (6.4)	2 (3.7)	0.005 ^d^
Cumulative live birth rate [*n* (%)]	23 (46.9)	23 (24.5)	13 (23.2)	0.009 ^d^

Data expressed as mean ± standard deviation. *p*-value < 0.05 was considered statistically significant. βHCG: beta human chorionic gonadotropin; COCs: cumulus–oocyte complexes; FSH: follicle-stimulating hormone; FET: frozen-embryo transfer; ^a^ The Kruskal–Wallis multiple comparison test was used to determine which group differed from others. ^b^ Comparison between low and high. ^c^ Comparison between low and middle. ^d^ Chi-square test. NS: not statistically different.

**Table 3:** Correlation between serum anti-Müllerian hormone (AMH) and in vitro fertilization (IVF) cycle characteristics and study outcomes. An asterisk (*) denotes statistically significant values.

	AMH *r* (Spearman’s Rank Correlation Coefficient)	*p*-Value
Age	−0.205	0.005 *
BMI	0.188	0.009 *
FSH, mUI/mL	−0.273	<0.001 *
LH, mUI/mL	0.188	0.011 *
LH:FSH ratio	0.275	<0.001 *
Total FSH dose, UI	−0.226	0.002 *
No. of COCs	0.091	NS
No. of follicles	0.171	0.025 *
Clinical pregnancy rate	−0.129	NS
Live birth rate	−0.114	NS
Cumulative live birth rate	−0.133	NS

BMI: body mass index; FSH: follicle-stimulating hormone; LH: luteinizing hormone; COCs: cumulus–oocyte complexes; NS: not statistically different.

## Data Availability

The data underlying this article will be shared upon reasonable request to the corresponding author.
